# First of All, I Feel Very Loved: Humanism and Cancer Care in South India

**DOI:** 10.1200/JGO.2016.006296

**Published:** 2016-08-31

**Authors:** Christopher M. Booth

**Affiliations:** Queen’s University, Kingston, Canada

As a senior medical resident I made a last-minute career detour into medical oncology after spending just 2 days in the cancer clinic. What drew me strongly to oncology were the unique and very real human interactions oncologists have with their patients. The opportunity to be present and bear witness to the courage of patients and families who face cancer is a true privilege. We repeatedly see patients let go of the peripheral things in life that do not matter and focus on things that matter most. Being part of this experience can be uplifting and inspiring. I knew that the relationship between oncologist and patient was something that I would treasure throughout my career.

Humanistic medicine is a contemporary movement that embraces the fundamental elements of medicine, including integrity, clinical excellence, compassion, altruism, respect, empathy, and service.^1^ Recent literature shows that almost half of medical oncologists experience burnout and that we are at risk of losing the ideals that bring us to medicine in the first place.^2^ In this commentary, I describe how a recent sabbatical in South India broadened my perspective on what matters most in the practice of medicine.

As a university-based medical oncologist and health services researcher, I spend my time caring for patients, undertaking clinical research, and teaching house staff. In 2014, I was invited to participate in the World Oncology Forum (WOF).^3^ This event brings together a diverse group of individuals from across the globe who have expertise in clinical care, research, education, advocacy, and health policy. As a health services researcher with an interest in optimizing the societal benefit of new cancer therapies,^4^ the WOF theme, “Treat the Treatable,” was highly relevant. This meeting allowed me to present my own work and interact with like-minded individuals from high-income countries and low- to middle-income countries (LMICs). This led to an opportunity to spend a 3-month sabbatical in South India to build a collaborative program in cancer health services research. In late 2015, my wife and I packed up our four young children and moved to Kerala, where I had the privilege of working at the Regional Cancer Centre (RCC) in Trivandrum.

Cancer is now recognized as a major threat to public health in LMICs.^5^ Access to cancer care demonstrates a striking paradox, in which 5% of global resources for cancer are spent in LMICs, where 64% of global deaths as a result of cancer occur.^5,6^ More than one million new occurrences of cancer are diagnosed annually in India, which has a population of 1.2 billion.^7^ The high cancer mortality rate (68% annual incidence) in India likely reflects the large proportion of patients who present with advanced disease and the limited access to cancer care in some parts of India.^8^

Kerala (population, 33 million) is located on the southwest coast of India and has the highest literacy rate, greatest life expectancy, and lowest infant mortality rate in India.^9,10^ Kerala is widely recognized as a leader among LMICs in the delivery of cancer care.^11-15^ The RCC in Trivandrum is the oldest and largest comprehensive cancer treatment center in Kerala. The RCC is a government institution that provides highly subsidized or free care to most of its 16,000 new patients seen each year.

The most striking observations from my experience were (1) the overwhelming number of sick patients, (2) the advanced stages of disease, (3) the compassion and dedication of Indian oncologists, and (4) the remarkable similarity in treatment delivered to patients at RCC and patients in Canada.

In Canada and the United States, a busy medical oncologist might see 25 patients in a full day of clinic. Unless we are on call, we do not see patients on the weekend. My colleagues at the RCC see patients 6 days per week, and it is not uncommon for physicians to see up to 80 patients per day. These high clinic volumes are not surprising when one considers that RCC has almost four times the patient volume of my own center yet has two thirds of the clinical work force. With this remarkably high patient volume, the most notable difference in care was the lack of time to have important conversations about prognosis, treatment options, and goals of care. My colleagues at the RCC recognized this limitation and addressed these important issues to the best of their ability in the midst of an overwhelming volume of sick patients. Although I could not follow many conversations that took place in Malayalam, the facial expressions and body language of patients and families in India are the same as what I see in my clinic at home in Canada. I saw evidence of fear, worry, and pain, but I also saw expressions of hope, optimism, and evidence of a deep caring for the sick. The dedication, skill, and compassion of my Indian colleagues cannot be overstated. Many of them could work in the private sector in India or elsewhere in the world, where they would have much higher income for comparatively less work. However, I was struck by their senses of altruism and individual responsibility to improve the lives of patients with cancer in India. These senses of duty, purpose, and altruism made it especially difficult to receive weekly e-mail bulletins from my province’s physician organization in Canada that was involved in a public conflict with the government over physician remuneration.

I was amazed that, despite the grueling workload on the other 6 days per week, I was repeatedly invited to large community events held on Sunday (the only day away from work), at which a team of physicians and community-based volunteers offered cancer awareness and screening camps to the most destitute communities. At one of these events, I was treated as a guest of honor and shared the stage with the mayor of Trivandrum. My children quickly learned that speeches at these events can go on for some time and are occasionally punctuated by explosions of loud firecrackers. When the time came for me to speak, I offered greetings “from the people of Canada in the Great White North” and thanked the community for so graciously welcoming my family. With their bright blond hair and blue eyes, my children were the subject of many photographs and became instant celebrities in Kerala.

The incredible efficiency of the RCC was made possible by the strong work ethic of my Indian colleagues and by the patients’ families, who play essential roles in the coordination and delivery of care. Each patient was accompanied by at least one family member, who took responsibility for bringing test results and consultant reports to clinic, booking future appointments, and even hand-delivering chemotherapy orders to the treatment unit. The importance of family support permeated all elements of cancer care that I witnessed in Kerala.

Outside of the RCC, I had the privilege of working with Pallium India, a globally recognized non-governmental organization that provides clinical care, educates health professionals, and serves as a national advocate for palliative care. During home visits with Pallium India, I witnessed firsthand how a community-based network of volunteers can provide comfort in the face of scarce resources. Traveling by van through the narrow streets of Trivandrum, I made house calls with a physician, nurse, and social worker. The breadth of medical, emotional, and psychosocial problems to which these teams tend is remarkable. I witnessed untold care and compassion delivered to a young boy dying at home with painful bone metastases. I witnessed the joy that the team’s visit brought to a socially isolated man with Parkinson disease. He remained a gracious host to us as visiting Canadians even after my resident forgot to latch the gate and his chickens almost escaped! I also met an impoverished mother and father who provided remarkable care to their cognitively impaired daughter in their clean but simple home with dirt floors. Although unable to afford diapers (which cost $2 per day), these parents managed to avoid pressure sores in their incontinent daughter by changing soiled bedsheets and washing them by hand several times per day. The compassion of the physicians and staff at Pallium India and the critical role of family in the provision of care for the terminally ill represent the epitome of humanistic medicine.

My family and I have returned to Canada, but we remain profoundly affected by our time in Kerala. Our children have a newfound appreciation of the many things we have in Canada for which we should be grateful. They also have gained insight into poverty and what life is like for people who live elsewhere in the world. I also have returned to work with a renewed sense of purpose. The work ethic, skill, and dedication of my colleagues in India was humbling and has helped me focus on how I can use my career as an oncologist and health services researcher in a high-income country to achieve the greatest good for patients worldwide. My sabbatical has also reaffirmed the role of humanism in my clinical work. Even when we cannot cure the disease, we can still provide comfort, care, and compassion to our patients. The most striking reminder of this came during a home visit of an elderly man who was dying with pancreatic cancer. I asked the physician with whom I was working to ask the patient what he most valued about the visits from the palliative care team. His response captured the essence of humanism in cancer care; translated from Malayalam, he stated simply, “First of all, I feel very loved.”
